# Progress in the study of functional genes related to direct seeding of rice

**DOI:** 10.1007/s11032-023-01388-y

**Published:** 2023-05-24

**Authors:** Xuezhong Li, Jingfang Dong, Wen Zhu, Junliang Zhao, Lingyan Zhou

**Affiliations:** 1 College of Agriculture and Biology, Zhongkai University of Agriculture and Engineering, Guangzhou, 510225 Guangdong China; 2 Rice Research Institute, Guangdong Academy of Agricultural Sciences/Guangdong Key Laboratory of New Technology in Rice Breeding/Guangdong Rice Engineering Laboratory, Guangzhou, 510640 China

**Keywords:** Direct seeding of rice, Functional gene, Seed vigor, Low-temperature tolerance germination, Low-oxygen tolerance growth, Early seedling vigor, Early root vigor, Lodging resistance

## Abstract

Rice is a major food crop in the world. Owing to the shortage of rural labor and the development of agricultural mechanization, direct seeding has become the main method of rice cultivation. At present, the main problems faced by direct seeding of rice are low whole seedling rate, serious weeds, and easy lodging of rice in the middle and late stages of growth. Along with the rapid development of functional genomics, the functions of a large number of genes have been confirmed, including seed vigor, low-temperature tolerance germination, low oxygen tolerance growth, early seedling vigor, early root vigor, resistance to lodging, and other functional genes related to the direct seeding of rice. A review of the related functional genes has not yet been reported. In this study, the genes related to direct seeding of rice are summarized to comprehensively understand the genetic basis and mechanism of action in direct seeding of rice and to lay the foundation for further basic theoretical research and breeding application research in direct seeding of rice.

## Introduction

Rice (*Oryza sativa* L.) is a major food crop for more than half of the world’s population (Zhang et al. 2014; Zeng et al. 2017). Efficient and sustainable development of the rice industry is a major challenge for food security (Hickey et al. 2019).

Traditional rice cultivation is generally performed via seedling transplantation, which provides suitable soil conditions for rice rooting and survival. At the same time, it provides a good environment for weed control (Singh et al. 2001). According to statistics, 77% of the world’s rice production is achieved by seedling transplantation, and this percentage is as high as 95% in China (Peng et al. 2009; Rao et al. 2007). However, owing to societal development and environmental impacts, the cultivation technology for seedling transplanting is facing increasingly serious problems in production (Farooq et al. 2011). First, with the social transformation and transfer of rural labor, the shortage of rural labor is becoming increasingly serious. The cultivation technology of seedlings and transplanting, which requires a lot of labor, does not meet the requirements of current social development. The second problem is related to water resources. The seedling transplanting cultivation technology requires a large amount of water for irrigation, resulting in the waste of water resources (Savary et al. 2005). Another problem is greenhouse gas emissions during rice production. Transplanting cultivation technology causes rice to soak in water during most of the growth stage, resulting in a large amount of greenhouse gas emissions owing to the oxygen-deficient environment (Hussain et al. 2020). According to statistics, rice paddy fields emit more than 30% of global methane gas (Gupta et al. 2021).

Considering the above-mentioned problems, direct seeding of rice cultivation technology has gradually gained attention. Direct seeding is a cultivation technology in which rice seeds are sown directly in the field, without going through the seedling stage. Currently, direct seeding can be classified into three types: dry direct seeding, wet direct seeding, and water direct seeding (Mahender et al. 2015). Dry direct seeding means sowing dry seeds directly into dry soil; wet direct seeding means sowing freshly sprouted rice seeds into moist soil; and water directing seeding means sowing germinated rice seeds in flooded fields. Previously, direct seeding of rice technology was widely used in the United States, Europe, and other developed countries with high levels of agricultural mechanization (Farooq et al. 2011). In recent years, with the vigorous development of science and technology, direct seeding of rice technology has received increasing attention, and many developing countries have begun to promote it on a large scale. In some countries, the area covered by direct-seeded rice has exceeded 50% (Farooq et al. 2011). Studies have shown that direct seeding can reduce total labor requirements from 11 to 66% depending on the season, location, and type of direct-seeded rice, and can significantly increase production efficiency compared with seedling transplanting (Chakraborty et al. 2017; Kumar and Ladha 2011). With improvements in water management conditions, nitrogen fertilizer utilization can reach 80% in direct-seeded rice (Wu et al. 2020). Greenhouse gas emissions can be reduced by delaying the initial flooding of direct-seeded rice, thereby reducing global warming (Kumar and Ladha 2011; Bhullar et al. 2016; Tao et al. 2016; Tirol-Padre et al. 2016). With proper water management patterns at the seedling stage of direct-seeded rice, seedling emergence and establishment rates can be improved, weed control can be improved, and tillering can be promoted, thus increasing the yield (Yadav et al. 2011; Li et al. 2017). Therefore, as labor costs continue to increase and the level of agricultural mechanization increases, direct seeding has become the main method of rice cultivation worldwide (Zhou et al. 2019).

Although direct seeding of rice technology has many advantages, its application and promotion still face significant problems and challenges. The common problems faced by direct seeding of rice are low seedling rate, serious weeds, and easy lodging of rice in the middle and late stages of growth (Chauhan and Abugho 2013; Setter et al. 1997). First, a low seedling rate is the primary factor that leads to a low and unstable yield of direct-seeded rice, which affects the starting number of seedlings directly and then affects the quality and final yield of the rice (Farooq et al. 2006). The main biological problems associated with low seedling rates include low seed germination and restricted early growth. If seed vigor, low-temperature tolerant germination, and low-oxygen tolerant growth can be improved by direct seeding, the whole seedling rate can be effectively increased (Wang et al. 2010; Zhang et al. 2014; Ismail et al. 2009). Previous reports have suggested that seeds with high vigor always have more uniform emergence than those with low vigor (Egli and Rucker 2012). Second, weeds affect more than 30% of the yield of direct-seeded rice and are another important factor limiting the yield of direct-seeded rice (Xu et al. 2019; Chauhan 2013). There are many types of weeds in rice fields, and the unequal distance between rice and weeds in a direct-seeded rice field provides space for the growth of all types of weeds. If rice seedlings have stronger seedling and root vigor, they can grow quickly and absorb more sunlight and nutrients than weeds, which in turn inhibits weed growth (Zhao et al. 2006; Finch-Savage et al. 2010). Susceptibility to lodging is also an important problem for direct-seeded rice (Setter et al. 1997). Because the rice seeds are sown on the soil surface of the paddy field, the roots are shallow. In the middle and late stages, the rice population is prone to poor ventilation and light penetration, the nutrients may be insufficient, the internodes at the base of the rice plants are too long, and the stalk walls become thin, resulting in easy lodging in the later stages, which not only affects the rice harvest and reduces rice production but also seriously reduces the quality of rice (Nguyen and Ferrero 2006; Kaur et al. 2017; Yadav et al. 2017).Therefore, rice varieties with high resistance to lodging are more suitable for direct seeding of rice.

However, selection and breeding of modern rice varieties have been carried out using conventional seedling transplanting technology. Many excellent functional genes, such as those involved in low-temperature germination tolerance, low-oxygen growth tolerance, and early seedling vigor, which are suitable for direct seeding, have not been effectively selected. As a result, most modern varieties are unsuitable for direct seeding of rice (Anandan et al. 2016). According to statistics, most of the current high-yielding varieties experience a 20–50% yield drop when applied to direct seeding (Heredia et al. 2021). Therefore, mining functional genes for the traits required for direct seeding and introducing them into existing high-yielding varieties using molecular marker-assisted selection is key to breaking through the bottleneck of the promotion and application of direct-seeded rice.

**Fig. 1:**
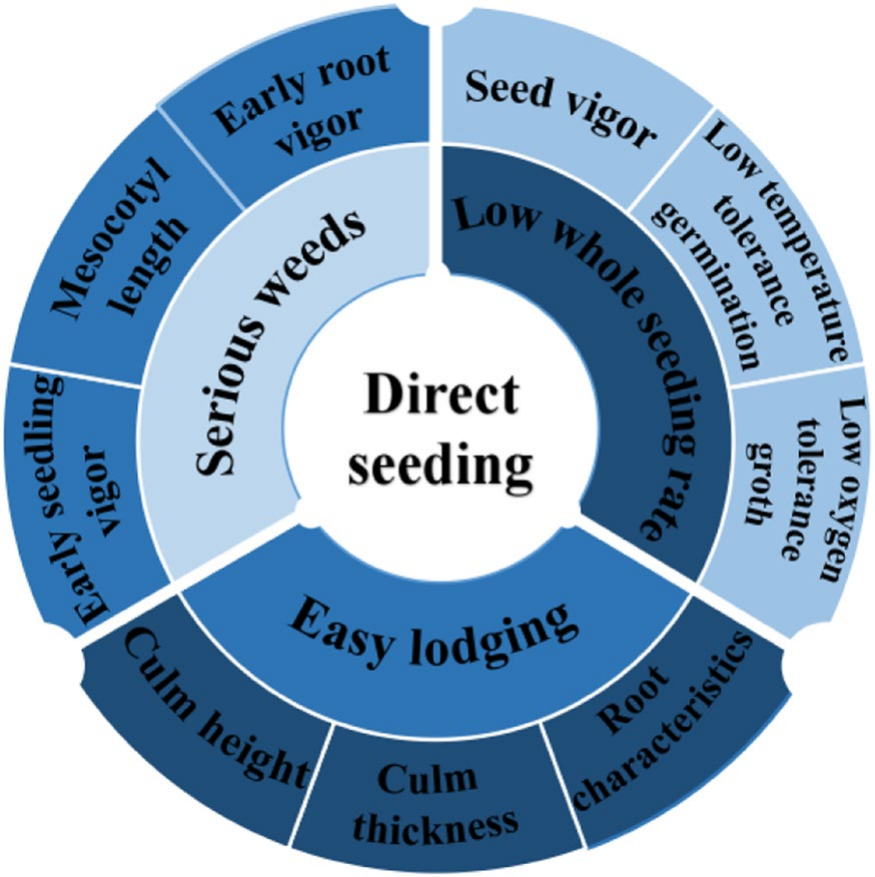
Three major problems and six traits faced by direct seeding of rice

In this study, we confronted the three major problems in direct seeding of rice: low whole seedling rate, severe weeds, and susceptibility to lodging in the middle and late stages of rice growth, and focused on six traits affecting the three major problems: seed vigor, low-temperature germination tolerance, low-oxygen growth tolerance, early seedling vigor, early root vigor, and lodging resistance (Fig. 1). We have reviewed and summarized the relevant functional genes and their mechanisms of action to comprehensively understand the genetic basis and mechanism of action in direct-seeded rice, and lay the foundation for further basic theoretical research and breeding application research in direct seeding of rice.

## Research progress on functional genes related to the whole seedling rate

### Functional genes and mechanisms of action related to seed vigor

Seed vigor is closely related to the seedling vigor (He et al. 2019a; Finch-Savage and Bassel 2016; Gommers and Monte 2018; Rajjou et al. 2012). Seed vigor not only affects seed germination and emergence ability (how many seedlings emerge, how fast, etc.), but also directly affects the amount of early seedling growth and resistance to adverse external conditions (Foolad et al. 2007; Wang et al. 2010). Thus, improving seed vigor is essential for direct seeding of rice (Mahender et al. 2015).

**Fig. 2:**
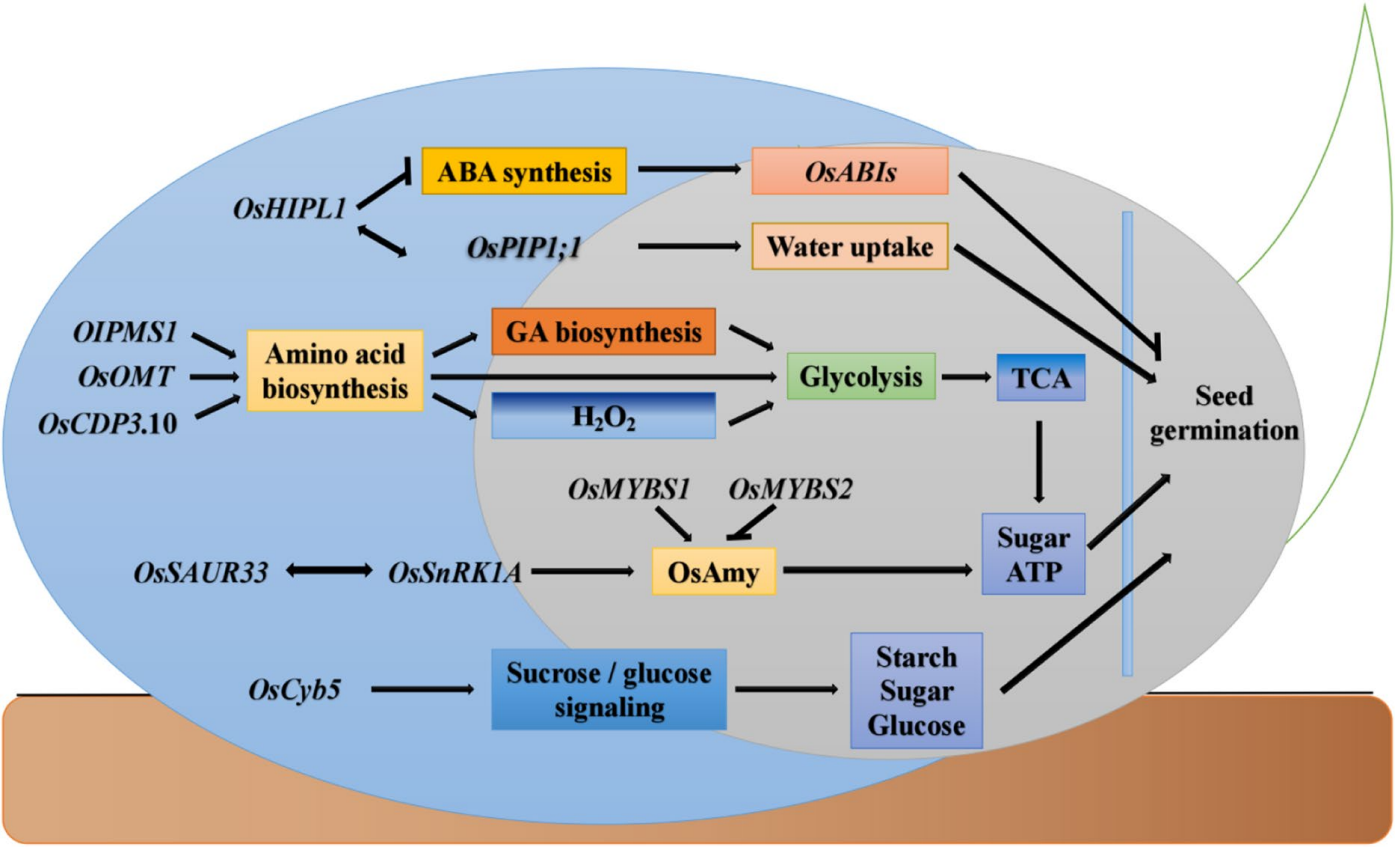
Functional genes and mechanisms of action related to rice seed vigor. Arrows and lines with slanted dashes indicate the positive and negative effects, respectively

Seed vigor is influenced by genetic and environmental factors during the seed development, storage, and germination stages (Zhao et al. 2021a). *OsIPMS1* is a functional gene that affects early seed vigor in rice and catalyzes leucine biosynthesis. The knockout of *OsIPMS1* results in a decrease in the amino acid content of rice seedlings and a significant reduction in rice seed vigor. Further studies have shown that *OsIPMS1* provides nutrition for seed germination and seedling growth by enhancing the biosynthesis of free amino acids during seed germination, promoting gibberellin synthesis, and enhancing the tricarboxylic acid cycle and glycolytic reactions during seed germination (He et al. 2019b). *OsSAUR33* can interact with *OsSnRK1A*, a regulator of the sugar signaling pathway, and thus regulate rice seed vigor (Zhao et al. 2021b). *OsCDP3.10* regulates rice seed vigor by affecting amino acid accumulation and stimulating the production of hydrogen peroxide (H_2_O_2_) during seed germination (Peng et al. 2021a). *OsHIPL1* is a novel gene that regulates rice seed vigor. In greenhouse experiments and field direct seeding trials, *OsHIPL1* was found to upregulate the expression of endogenous ABA synthesis-related genes (*OsZEP* and *OsNCED4*), downregulate the expression of catabolism-related genes (*OsABA8ox3*), and significantly increase endogenous ABA content, but also regulate water uptake by affecting the hydropore protein *OsPIP1;1* during seed germination and regulation of rice seed vigor (He et al. 2022). *OsOMT* affects seed vigor by regulating amino acid synthesis, glycolysis, and the tricarboxylic acid (TCA) cycle. Mutations in *OsOMT* reduce the levels of amino acids involved in energy production during seed germination, which may reduce energy availability and lead to low seed vigor in *Osomt* mutants (Li et al. 2021). *OsCyb5* affects seed vigor mainly by altering starch and sugar mobilization and glucose accumulation in germinating seeds. Compared with the wild plants, the *Oscyb5* mutant shows no significant differences in grain length, width, thickness, thousand-grain weight, starch content, protein content, and soluble sugar content, but shows significantly lower starch and sugar mobilization as well as glucose content during the germination stage (Huang et al. 2021). These results provide important clues for amino acid seed initiation treatment and molecular breeding selection to improve seed vigor in direct seeding of rice (Fig. 2).

### Functional genes and mechanism of action of low-temperature germinability in rice

Low-temperature stress (LTS) is a major abiotic stress that affects rice growth and ultimately decreases grain yield (Zhang et al. 2014; Arshad et al. 2017). Owing to its tropical or subtropical origin, rice is generally susceptible to low temperatures at many stages, including germination, seedling growth, and panicle initiation. In the germination stage, the optimum temperature of cultivated rice is 25–35 °C, but the temperatures are frequently below 15 °C during the sowing period in direct seeding in the early season, which leads to poor germination and weak seedling establishment, and subsequently a decrease in yield (Luo 2011; Zhang et al. 2014). This is a major factor that limits the application of direct-seeded rice (Yang et al. 2020).

**Fig. 3:**
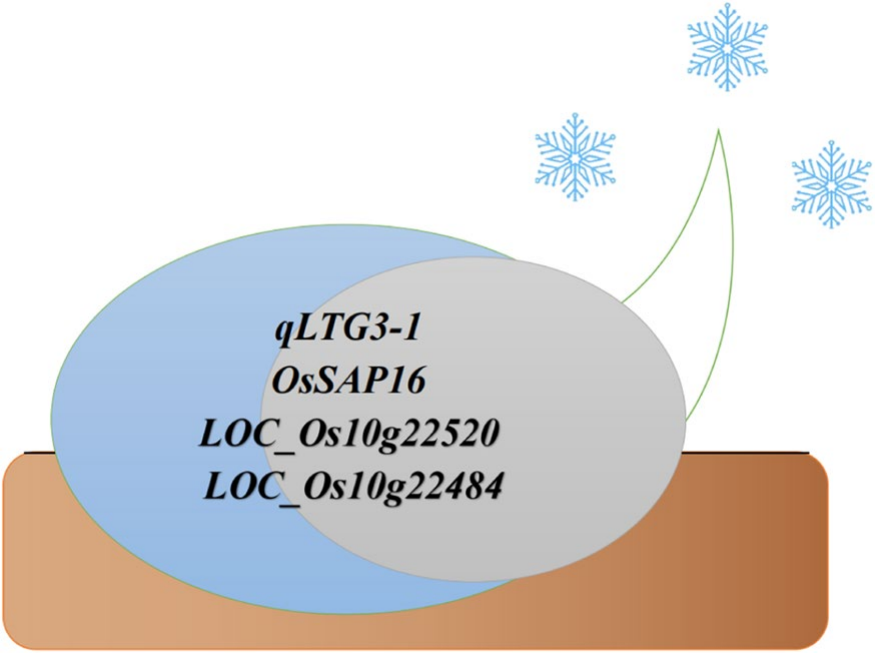
Relevant genes under low-temperature germination in rice

Low-temperature germinability (LTG) is a complex trait that significantly varies among rice cultivars. To date, more than 200 QTLs have been mapped in rice (Shirasawa et al. 2012; Biswas et al. 2017; Najeeb et al. 2021). However, many QTLs have relatively small effects, explaining less than 20% of phenotypic variance (Shim et al. 2020). Furthermore, only two functional QTLs have identified causal genes. *qLTG3-1* is the first characterized gene that confers low-temperature germination. *qLTG3-1* is a major QTL that confers over 30% phenotypic variance (Fujino et al. 2004). The causal gene *qLTG3-1* encodes a protein with an unknown function and is possibly involved in tissue weakening (Fujino et al. 2008). Further studies demonstrated that *qLTG3-1* tolerance contributes to several types of stress at the seed germination stage, including low and high temperature, high salt, and high osmotic conditions. This may be related to the pleiotropic effects of *qLTG3-1* in rice growth (Fig. 3) (Fujino and Sekiguchi 2011).

*OsSAP16* is the second functional gene identified to contribute to LTG variation in rice. *OsSAP16* was also the first to identify an LTG gene in a genome-wide association study (GWAS). *OsSAP16* encodes a stress-associated protein containing two AN1-C2H2 zinc-finger domains. After GWAS analysis of LTG using 187 accessions, *OsSAP16* was identified as a candidate QTL gene. Further functional analysis indicates that a higher expression of *OsSAP16* can increase LTG in rice (Fig. 3) (Wang et al. 2018b).

A total of 11 LTG QTL were identified using GWAS in our previous study, and two candidate genes, *LOC_Os10g22520* and *LOC_Os10g22484*, were identified in the QTL on chromosome 10 by combining the GWAS and genome-wide expression profiling results (Yang et al. 2020). *LOC_Os10g22520* encodes glycoside hydrolase (GH, EC3.2.1), a class of enzymes that hydrolyzes glycosidic bonds and plays an important role in the hydrolysis and synthesis of sugars and glycoconjugates in living organisms. Glycosidic hydrolases have been reported to be involved in the degradation of cell wall polysaccharides and in controlling the loosening of plant cell walls and thus regulating germination, growth and development, fruit ripening, abscission and cell adhesion (Minic and Jouanin 2006; Minic 2008). *LOC_Os10g22520* exhibited significantly higher expression levels in the high LTG lines than in the low LTG lines, suggesting that it may be more actively involved in the degradation of cell wall polysaccharides and cause tissue weakening or involvement in carbohydrate mobilization to drive the growth of germinating embryos in high LTG lines, which consequently enhances their germination at low temperatures. *LOC_Os10g22484* encodes an NBS-LRR domain-containing protein and it is possible that sequence variation in the NBS-LRR domain-containing protein gene *LOC_Os10g22484* might result in increased germination at low temperatures in rice (Fig. 3).

### Functional genes and mechanism of action of low-oxygen tolerance growth

The three principal modes of direct-seeded rice are dry, wet, and water (Mahender et al. 2015). In dry and wet direct seeding, rice seeds may encounter flooded conditions due to uneven soil leveling, heavy rainfall, or poor drainage. In water direct seeding, seeds are germinated and grown under submerged conditions for a short time. It is critical for rice seeds to achieve rapid and uniform germination and seedling establishment under low-oxygen and submergence conditions (Yu et al. 2021). Therefore, tolerance to low-oxygen conditions at these early stages is a prerequisite for effective direct-seeded rice in rainfed and flood-affected areas. Rice has evolved various survival strategies under hypoxia or anoxia conditions (Lee et al. 2009). Generally, they can be divided into two types: flood-tolerance mechanisms and flood-avoidance mechanisms (Yu et al. 2021).

#### Functional genes and mechanism of action related to germ elongation under low-oxygen conditions in rice

**Fig. 4:**
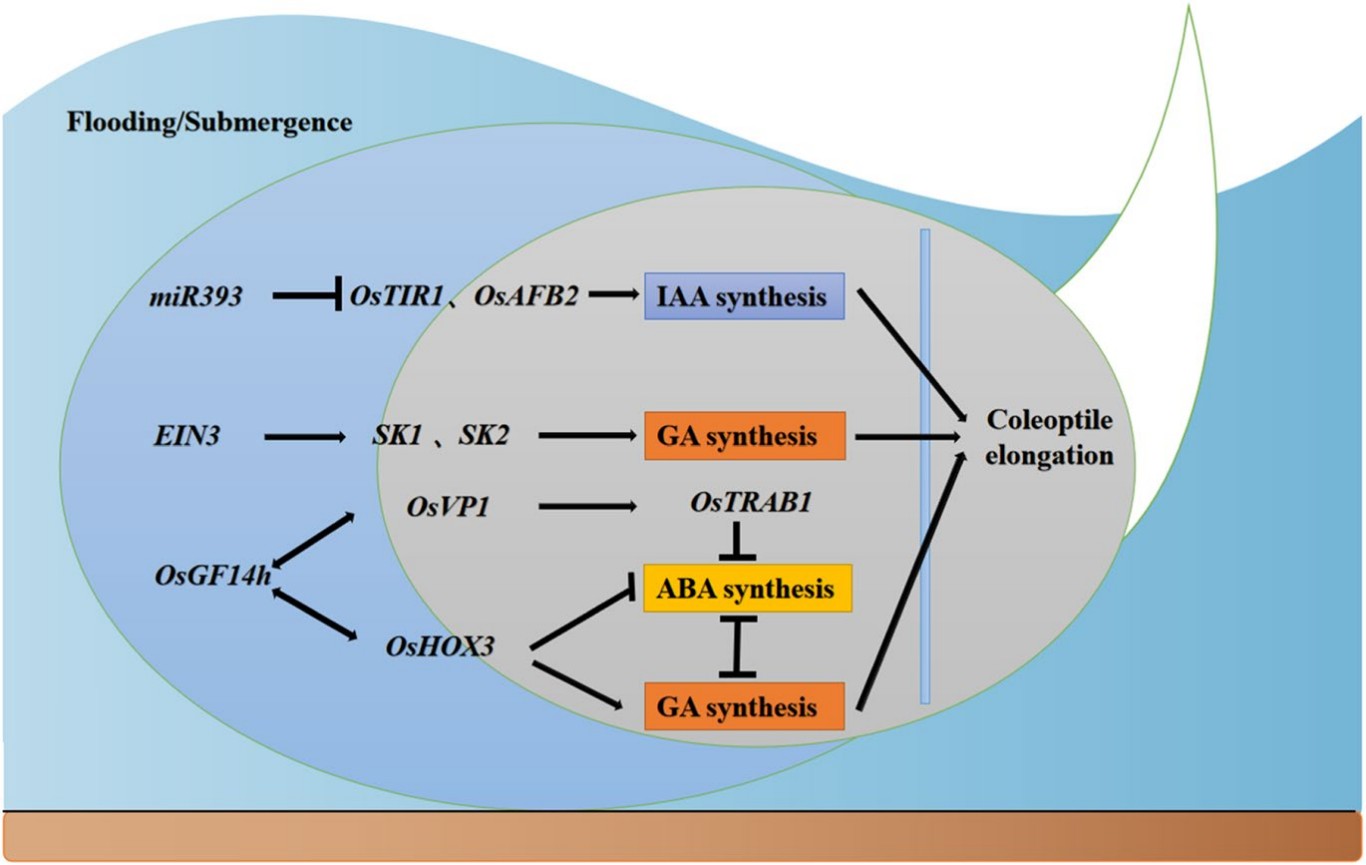
Functional genes and mechanisms of action related to the low O_2_ escape strategy of rice seed germination under submerged conditions. Arrows and lines with slanted dashes indicate positive and negative effects, respectively

The coleoptile of germinating rice seeds elongates to protect the true leaves during emergence from the soil and provide nutrients for developing tissues (Pucciariello 2020). Under submergence, the coleoptile acts like a snorkel that reaches the water surface and connects with air (Narsai et al. 2015). The hollow structure of the coleoptile allows the seeds to access oxygen and establish aerobic respiration to meet the needs of seedling growth. This is an effective strategy for achieving anaerobic germination and growth in rice (Kordan 1974) (Fig. 4).

Several functional genes and small RNAs that regulate rice germ elongation under hypoxic conditions have been identified. Under hypoxia, the expression of the small molecule RNA *miR393* is suppressed, which in turn contributes to the expression of the regulatory growth hormone receptor genes *OsTIR1* and *OsAFB2.* This weakens the inhibitory effect of *miR393* on the growth hormone signaling pathway, which in turn promotes the transcription of downstream endosperm elongation-related genes (such as *OsEXPA4*, *OsEXPA7,* and *OsEXPB12*) through the IAA pathway, thereby promoting endosperm elongation (Bian et al. 2012; Xia et al. 2012; Si-Ammour et al. 2011; Lasanthi-Kudahettige et al. 2007; Guo et al. 2016). In addition to growth hormones, ethylene and gibberellin are phytohormones that have important effects on the growth of the endosperm sheath. Under flooded conditions, low concentrations of ethylene can act as signaling molecules, forming a trimeric response (CTR) with the ethylene receptor (ETR) on the cell membrane and inducing the differential expression of ethylene insensitive (*EIN*) and ethylene insensitive like (*EIL*) genes (Kazan 2015). The *EIN/EIL* protein binds to the promoter region of the ethylene response factor (*ERF*) gene to initiate expression of *ERF* family genes (Alexander 2002).

Using deep-water rice as the study target, Hattori et al. (2009) found that two ERF-VII-like transcription factors, *SNORKEL1* (*SK1*) and *SNORKEL2* (*SK2*), play key roles in the adaptation of deep-water rice to flooded environments. *SK1* and *SK2* encode ethylene response factors, and ethylene activates the ethylene signaling pathway when water submergence causes an increase in ethylene content. *EIN3* binds to the promoters of *SK1* and *SK2* to promote the expression of both, activating the gibberellin response required for stem elongation and regulating stem elongation in deep-water rice under submergence (Ayano et al. 2014), which in turn allows the plant to elongate rapidly underwater, enabling the plant to break through the water surface early and obtain oxygen, ultimately allowing it to survive under submerged conditions.

*SEMIDWARF1* (*SD1*) gene encodes *GA20ox,* a key enzyme in gibberellin synthesis. The Sd1 protein drives the increased synthesis of gibberellins, mainly GA4, and promotes internode elongation. *Sd1* expression allows rapid elongation of rice internodes after the 10-leaf stage in deep water, helping rice leaves emerge quickly and obtain oxygen (Kuroha et al. 2018). *OsGF14h* interacts with the transcription factors *OsHOX3* and *OsVP1*, which act as signaling switches to balance ABA signaling and GA biosynthesis. It can increase the seeding rate from 13.5% to 60.5% for anaerobically sensitive varieties under submerged direct-seeded conditions (Sun et al. 2022).

#### Functional genes and mechanisms of action related to metabolism and energy production in rice under hypoxic conditions

**Fig. 5:**
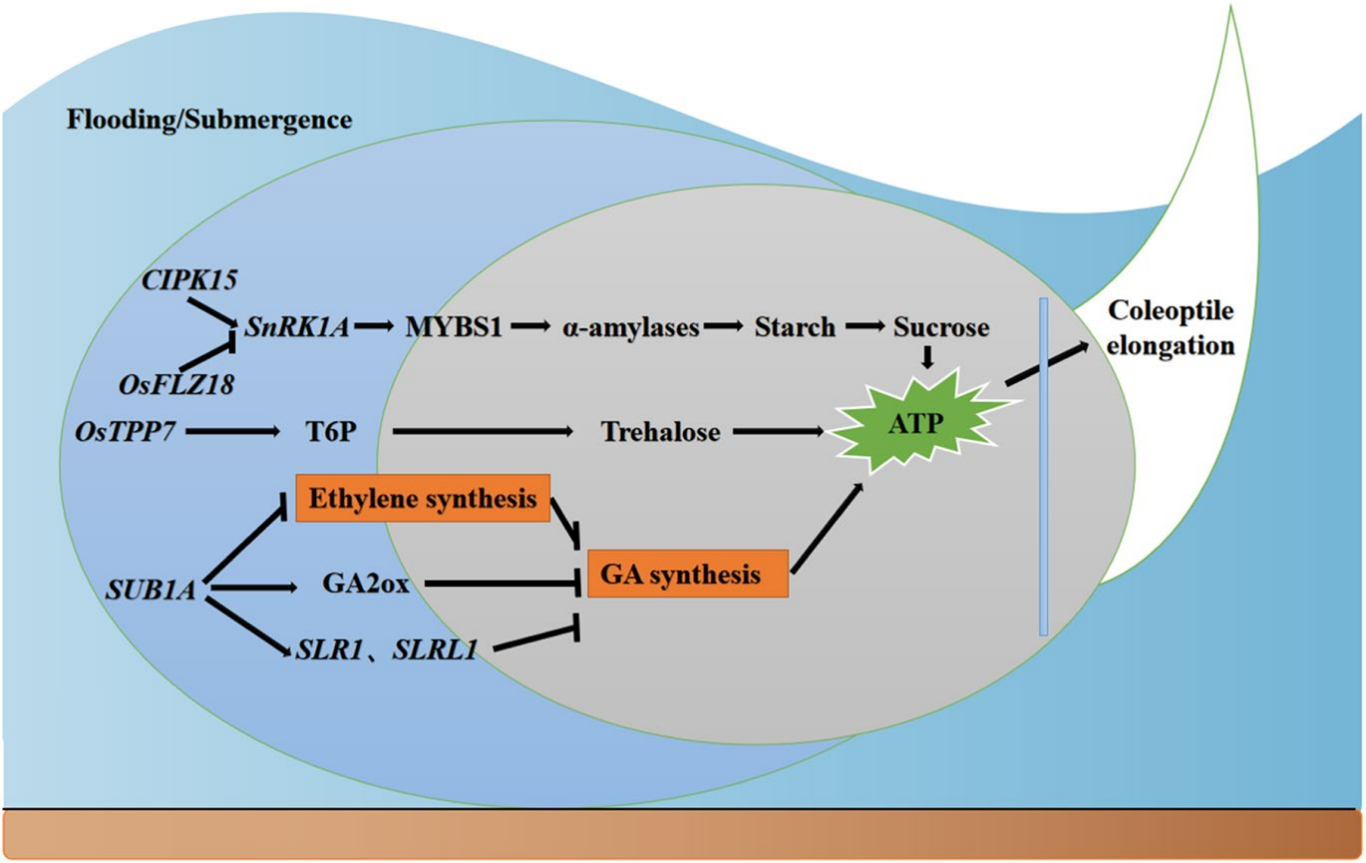
Functional genes and mechanisms of action related to low O_2_ quiescence strategy for rice seed germination under submerged conditions. Arrows and lines with slanted dashes indicate the positive and negative effects, respectively

Because of oxygen deficiency, aerobic respiration is severely inhibited in submerged germinating seeds, which shifts metabolic processes to anaerobic respiration. It has been shown that varieties tolerant to low-oxygen stress can germinate and grow faster and maintain their ability to use stored starch reserves through higher amylase activity and anaerobic respiration (Perata et al. 1992; Guglielminetti et al. 1995; Ismail et al. 2009). In starch metabolic pathways, α-amylase is one of the most abundant hydrolases and is involved in the mobilization of stored starch in rice. Among all α-amylases in rice, *RAmy3D* is induced by sugar starvation and O_2_ deficiency, which are important players in the fermentative metabolism pathway for metabolic shift regulation and energy production in response to submergence stress (Loreti et al. 2003; Hwang et al. 1999). This is another effective strategy for achieving anaerobic germination and growth of rice (Fig. 5).

To adapt to metabolic pattern shifting, gene expression was profoundly changed to activate essential mechanisms. In rice, anaerobic germination is regulated by sugar starvation and hypoxia-dependent Ca^2+^ signaling. Low oxygen levels and starvation activate α-amylases to hydrolyze starch and supply energy. Calcineurin B-like protein (CBL) interacting protein kinase 15(CIPK15), together with a CBL Ca^2+^ sensor, contributes to the decoding of Ca^2+^ signaling (Lee et al. 2009). *CIPK15* then activates *SnRK1A* and *MYBS1* to activate the downstream α-amylases. Simultaneously, large amounts of alcohol dehydrogenase are produced to ferment sugars to produce energy (ATP) so that the seeds have sufficient carbohydrates and energy to germinate in water. In this cascade, *SnRK1* kinase is the key regulator of rice energy balance (Crepin and Rolland 2019). Ma et al.’s (2021) genome-wide identification of FCS-like zinc finger (FLZ) proteins revealed that several members can interact with *SnRK1A*. Further transformation analysis indicated that one of the members of the rice FLZ family, *OsFLZ18,* interacts with SnRK1A and inhibits the transcriptional activation activity of *SnRK1A* in modulating the expression of its target gene *αAmy3*, thus modulating rice seed germination and coleoptile elongation under submergence conditions. *OsTPP7* is a functional gene cloned from an anaerobic germination QTL (*qAG-9–2*) in rice. *OsTPP7* encodes alglucose-6-phosphate phosphatase, which plays an important role in the metabolism of alglucose-6-phosphate. It has been shown that the *OsTPP7* gene ensures an energy source by regulating the source pool balance and increasing the sugar content delivered to the embryo as well as the germinal sheath, thus enabling rice germination survival under flooding stress (Kretzschmar et al. 2015). *SUB1A* is another functional gene that promotes anaerobic growth of rice. *SUB1A* inhibits plant tissue elongation by inducing the accumulation of gibberellin (GA) suppressors *SLR1* and *SLRL1*. *SUB1A* can inhibit ethylene synthesis and reduce the rate of chlorophyll degradation in plants through the jasmonic acid (JA) pathway for adaptation to flooded environments. *SUB1A* can induce the expression of *GA2ox*, which degrades GA in rice and leads to lower energy usage by suppressing rice growth (Niroula et al. 2012).

## Advances in the study of functional genes that compete with weeds effectively

### Functional genes and mechanisms of action related to early seedling vigor in rice

Early seedling vigor in rice is a key factor for the success of direct seeding (Anandan et al. 2020; Mahender et al. 2015). Seedling vigor refers to the overall expression of various active intensities and seed characteristics during germination and emergence. Seedling vigor affects later growth and development of the plant, and it has the ability to adapt to the surrounding environment, resistance to stress, crop yield, and quality (Mahender et al. 2015). Higher seedling vigor ensures rapid emergence of seedlings from the soil during direct seeding and efficient use of storage reserves for rapid root and shoot expansion, which could make rice compete with weeds and effectively suppress weed growth (Rao et al. 2007; Chauhan et al. 2015; Singh et al. 2017).

**Fig. 6:**
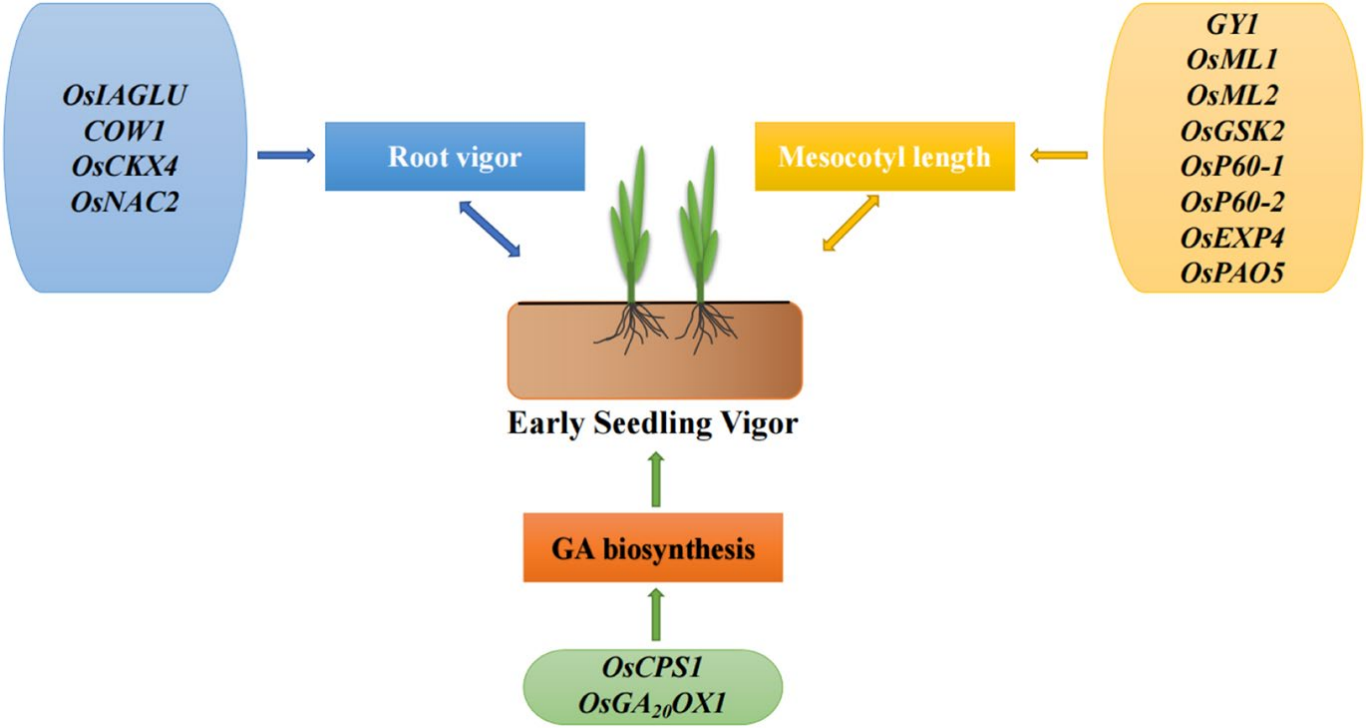
Genes related to early seedling vigor and their pathways of action

With the promotion of direct seeding technology, early rice vigor, including seedling and young root vigor, has been increasingly emphasized (Fig. 6). Early rice vigor is a quantitative trait that is controlled by multiple genes and influenced by various complex environmental factors. In recent years, several QTLs for rice seedling vigor have been identified. For example, Chen et al. (2019) identified 42 QTLs associated with early seedling vigor in rice, using 744 international rice seed resources; Xie et al. (2014) localized eight seedling vigor-related QTL using a recombinant self-incompatible line population and narrowed the localization interval of two of the main effective QTLs, *qSV-1* and *qSV-5c*, to 1.13 Mb and 400 kb; Dang et al. (2014) configured 15 biparental hybrid cross combinations to improve seedling vigor. These results provide a genetic basis for studies of rice seedling vigor and molecular breeding. However, only a few functional genes have been cloned to date (Zhang et al. 2017).

*OsGA20ox1* is the first gene to be associated with early seedling vigor. It encodes GA20 oxidase, a key enzyme that catalyzes gibberellin biosynthesis. The variation in the promoter sequence of *OsGA20ox1* can lead to elevated GA content in rice, thereby increasing rice seedling vigor (Abe et al. 2012). Ma et al. (2022) identified a GA synthesis gene, *OsCPS1*, by GWAS analysis of seedling length in internationally diverse rice species. It encodes ent-copalyl diphosphate synthase (CPS), the first key enzyme to formally enter the gibberellin biosynthetic pathway, which catalyzes the conversion of geranylgeranyl diphosphate (GGDP) to the bicyclic intermediate ent-copalyl diphosphate (CDP). After the mutation of *OsCPS1*, the plants were unable to produce any GA. Rice seedlings exhibits reduced vigor and even die (Otomo et al. 2004; Prisic et al. 2004; Sakamoto et al. 2004). GA synthesis and signaling pathways play important roles in regulating seedling vigor in rice.

Mesocotyl length is another important indicator of early seedling vigor, and is the main driver of the rapid emergence of rice seedlings from the soil (Zhan et al. 2020). Rapid mesocotyl elongation at the germination stage is beneficial for increasing seedling emergence speed and neatness, which could establish favorable conditions for direct seeding of rice at deep sowing (Dilday et al. 1990; Redoña and Mackill 1996; Lee et al. 2017; Zhan et al. 2020). Zhao et al. (2018) identified two master effector genes (*OsML1* and *OsML2*) that control mesocotyl elongation in rice, and a superior haplotype combination of *OsML1* and *OsML2* increased mesocotyl length by 4 cm, resulting in 85% high seedling emergence in soils with 10 cm soil cover.

Xiong et al. (2017) cloned *GY1*, a gene that regulates mesocotyl and radicle elongations. *Gy1* encodes a PLA1-type phospholipase localized in the chloroplast, which is the first enzyme in the jasmonic acid (JA) biosynthetic pathway. *Gy1* mutations result in the inhibition of PLA1 activity, which inhibits jasmonic acid biosynthesis and promotes elongation of the mesocotyl and radicle.

Sun et al. (2018) demonstrated that *OsGSK2*, a key component of the Brassinosteroids (BRs) signaling pathway, regulated mesocotyl length by GWAS analysis of rice mesocotyl length. BR inhibits phosphorylation of the U-type cell cycle protein CYCU2 by *OsGSK2* and ultimately promotes mesocotyl elongation.

Rice *OsP60-1* and *OsP60-2* promote rice mesocotyl elongation by promoting microtubule protein depolymerization and stimulating an increase in endogenous GA content (Liang et al. 2016). A gene that affects cell wall elongation, *OsEXP4*, influences mesocotyl elongation by affecting the length of the rice mesocotyl cells (Choi et al. 2003). The polyamine oxidase gene (*OsPAO5*) is another functional gene that controls rice mesocotyl elongation. Knockdown of the *OsPAO5* gene results in a significant increase in mesocotyl length and neater emergence at the early seedling growth stage, as well as an increase in yield traits, such as grain weight and grain number. *OsPAO5* is a target gene with the potential to improve direct seeding of rice performance (Lv et al. 2021). In addition, plant growth hormones are also an important factor affecting rice mesocotyl elongation under direct seeding conditions. Wang et al. (2021b) found that gibberellin-initiated treatments provided energy for mesocotyl elongation and deep seeding in direct seeded rice by activating the expression of genes related to starch and sucrose metabolism (*AMY2A* and *AMY1.4*) and thus increasing amylase activity and soluble sugar content under deep seeding conditions.

### Functional genes and mechanisms of action related to early root vigor in rice

In addition to seedling vigor, root vigor, which refers to the rapid establishment of a root system that can supply additional nutrients and water to the seedling, is also important for the success of direct seeding (Anandan et al. 2016; Mahender et al. 2015; Wang et al. 2018a). Seed nutrients can support germination during the short period of early growth. For example, nitrogen and iron in seeds may support seedling growth for 7–10 days, and phosphorus can support 10–14 days of growth (Wang et al. 2018a). Therefore, early root vigor is essential for rice cultivation to ensure a good crop stand, adequate uptake of water and nutrients, and competition for weeds (Anandan et al. 2022). Furthermore, stronger vigor of young root growth can also promote the anchoring of rice plants and improve lodging resistance, which is another major problem in rice direct seeding (Shah et al. 2019).

The genetic basis of root growth has been studied extensively in many reports. Using traditional bi-parental mapping approaches, many quantitative trait loci (QTLs) for early root growth traits have been identified in rice, including those related to maximum length, thickness, number, and mass (Courtois et al. 2009; Uga et al. 2012; Hanzawa et al. 2013; Kitomi et al. 2015; Li et al. 2015; Catolos et al. 2017). Yang et al. (2021) applied a root image analysis system to measure root growth diameter, including root length, root surface area, and root volume. Recently, GWAS have been implemented to detect loci associated with early root vigor. For example, Zhao et al. (2021c) examined the genetic architecture of variation in primary root length using a diverse panel of 178 accessions. They identified four QTLs for root length and further characterized *OsIAGLU*, which encodes a glucosyltransferase and regulates root length by modulating multiple hormones in the roots, including auxin, jasmonic acid, abscisic acid, and cytokinin.

It is well known that hormones such as auxin and cytokinin (CTK) play important roles in root development in rice. In addition to the functional genes related to root growth traits in rice, the mechanisms underlying hormones that participate in root development have also been investigated. For example, a rice mutant of the IAA biosynthesis gene *COW1* leads to a lower root-to-shoot ratio (Woo et al. 2007). Overexpression of the cytokinin oxidase/dehydrogenase gene, *OsCKX4,* improves root growth (Gao et al. 2014). The *OsNAC2* gene is involved in the regulation of growth hormone anabolism and signaling pathways. It promotes the expression of indoleacetic acid-amidated synthase genes *OsGH3-6* and *OsGH3-8* while repressing the expression of the growth hormone response factor *OsARF25*, leading to a reduction in growth hormone content in the root system and inhibition of rice root elongation. *OsNAC2* can also bind to the promoter of the cytokinin-degrading enzyme gene *OsCKX4* and repress its expression, which leads to an increase in the cytokinin content in the plant and inhibits rice root elongation (Mao et al. 2020).

## Progress in the study of functional genes related to lodging resistance

### Factors related to lodging resistance in rice

Lodging is related to stem bending, breakage, and root lodging in plants (Mulsanti et al. 2018; Zuo et al. 2017) and is one of the most concerning problems faced by farmers worldwide (Kuai et al. 2015). Lodging is one of the most important factors for grain yield potential in direct-seeded rice because it causes yield loss, lowers grain quality, and reduces the efficiency of mechanical harvesting (Wang et al. 2021a). Lodging has been found to be more severe in direct-seeded rice than in transplanted rice (Setter et al. 1997). Lodging, which is influenced by many interacting agro-morphological traits including genetic factors, planting methods, water management, and environmental factors, is a complex trait in rice (Yadav et al. 2017).

The short stature of plants is generally believed to be the most relevant to lodging resistance in rice. For example, the short stature of rice is the main target for improving lodging resistance, as well as the harvest index in rice, which is one of the most important landmark achievements in rice improvement over the past 50 years (Keller et al. 1999; Peng and Khush 2003). In 1959, Yaoxiang Huang, the father of semi-dwarf rice in China, from Guangdong Academy of Agricultural Sciences, bred the world’s first semi-dwarf indica rice variety Guang-chang-ai, which initiated rice-dwarfing breeding (Peng et al. 2021b). The rice-dwarfing breeding has resulted in a significant increase in lodging resistance in rice.

Culm diameter and thickness are other major factors contributing to lodging resistance in rice (Ookawa and Ishihara 1992; Zuber et al. 1999; Zhu et al. 2008; Ookawa et al. 2010). It has been reported that a greater culm diameter is strongly associated with culm wall thickness, which is an important factor in improving resistance to lodging in rice (Shah et al. 2019). Increasing lodging resistance by improving culm diameter is another method to breeding cultivars with high lodging resistance, long culms, and high biomass. Nomura et al. (2019) developed a rice variety with long and thick culm, and showed high yield and superior lodging resistance.

Cultivation techniques are also important factors influencing lodging resistance in direct-seeded rice (Shrestha et al. 2020). It has been reported that shorter basal internodes, better stem diameter, stem wall thickness, and a lower lodging index are found in dry direct seeding, in contrast to water and wet direct seeding (Wang et al. 2021a). Pan et al.’s (2019) study showed that improved nitrogen management could enhance lodging resistance and that lower internodes play a key role in the lodging resistance of rice. Further analysis demonstrated that changes in the expression patterns of genes related to cell wall loosening, lignin, and starch synthesis might be the underlying molecular mechanisms of this phenomenon.

### Functional genes and mechanism of action of rice resistance to lodging

As discussed above, lodging resistance is a complex quantitative trait that is affected by many traits in rice, including culm morphology, culm diameter and length, and cellulose content. Therefore, moderate plant height, large stem diameter, thick stem walls, and high lignin deposition have been recommended as the preferred traits to improve lodging resistance in direct-seeded rice (Yadav et al. 2017). Many lodging-related genes have been successfully cloned into rice, including *sd1, SBI, APO1, Gn1a/OsCKX2,* and so on. (Asano et al. 2007; Shah et al. 2019; Liu et al. 2018a; Ookawa et al. 2010; Tu et al. 2022).

The *sd1* gene, which confers semi-dwarfism to rice varieties, was the first gene utilized in several breeding programs to decrease plant height and substantially improve lodging in rice (Asano et al. 2007; Shah et al. 2019). Liu et al. (2018a) found that the GA2 oxidase encoded by the *SBI* gene could convert active gibberellins into inactive gibberellin compounds. The highly active *SBI* locus resulted in a significant reduction in active gibberellin content in the basal nodes of rice stalks, thereby inhibiting elongation of the basal internodes. A series of new rice varieties with a reasonable stalk node length structure and high resistance to lodging has been produced using *SBI* allelic mutations.

**Fig. 7:**
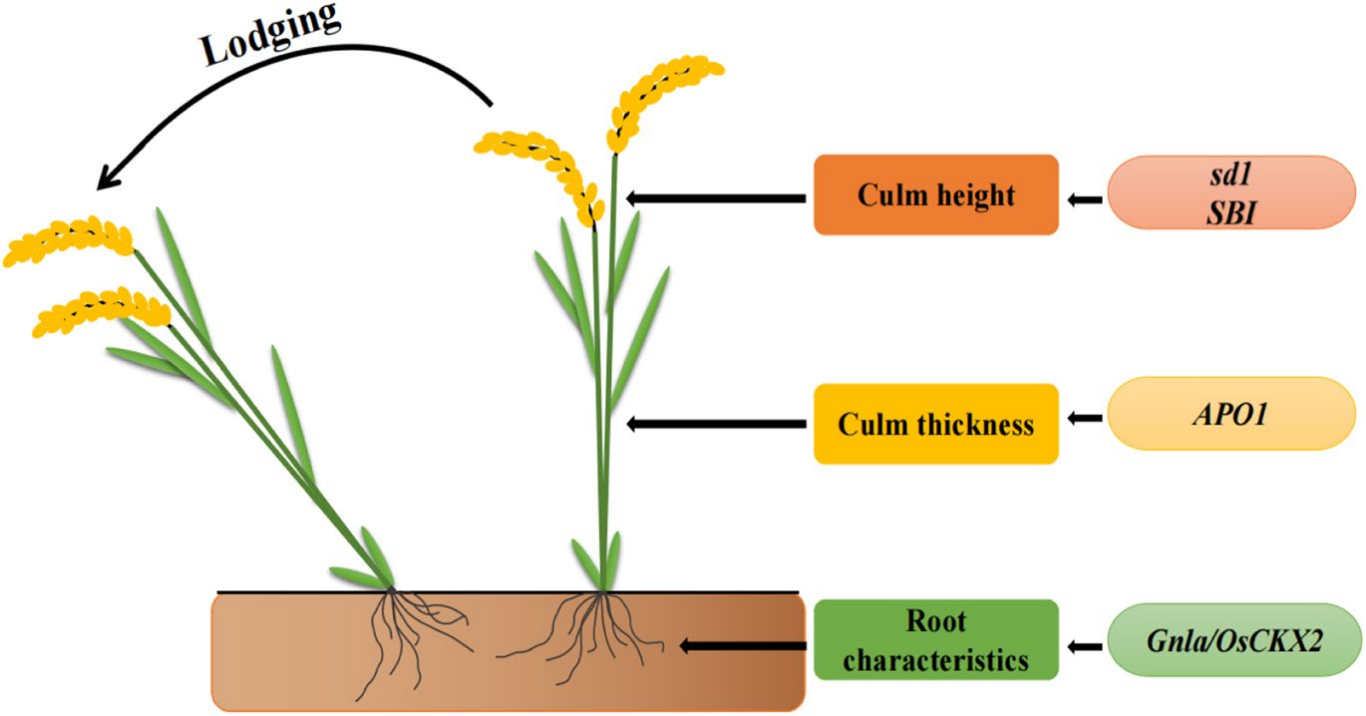
Genes related to lodging resistance and their action pathways

However, lodging still occurs in semi-dwarf varieties. This has prompted many studies to dissect genetic factors other than plant stature that confer lodging resistance in rice. For instance, the composition of the secondary cell wall in the culm is an important factor in determining lodging resistance because it is a rigid, thickened structure that determines the mechanical strength of the plant body (Zhang et al. 2016; Liu et al. 2018b). *APO1* encodes an F-box protein consisting of 429 amino acids, and is the first gene cloned to improve stalk strength. In addition, *APO1* can increase the number of grains per spike; therefore, *APO1* can be used to improve both yield and lodging resistance in rice (Ookawa et al. 2010). Root system characteristics, which could provide structural support to aerial organs by anchoring the plant to its growth substrate, are another important factor for lodging resistance (Shah et al. 2019; Meng et al. 2019). Recently, Tu et al. (2022) reported that the loss of the rice gene encoding cytokinin oxidase (OsCKX2) confers excellent lodging resistance. Loss-of-function of *Gn1a/OsCKX2* led to cytokinin accumulation in the crown root tip and accelerated the development of adventitious roots (Fig. 7).

## Conclusions and prospects

This review focused on three main problems facing direct seeding of rice and six related important traits. Functional genes and their mechanisms of action were reviewed and organized. This review is expected to lay the foundation for more in-depth basic theoretical research and breeding application research on direct-seeded rice.

However, many modern high-yielding and high-quality varieties are selected based on the cultivation method of transplanting, resulting in a large number of excellent functional genes for direct seeding-related traits that have been lost in modern varieties. Direct seeding-related traits are complex traits controlled by multiple genes, the effect of individual genes is small, and their expression is easily influenced by environmental and gene interactions. Therefore, mining related genes and trait improvements is very difficult. Researchers need to use germplasm resources with richer diversity, including wild rice resources, and modern technologies such as functional genomics, histology, and genome-wide association analysis to clone superior functional genes and dissect their molecular mechanisms. Simultaneously, using efficient and accurate modern molecular breeding techniques, the identified functional genes can be applied to the genetic improvement of direct-seeded rice. The process of selecting and breeding new rice varieties suitable for direct seeding will accelerate meeting the demand for direct seeding in rice production as soon as possible.

## Data Availability

Data sharing not applicable to this article as no datasets were generated or analyzed during the current study.

## References

[CR1] Abe A, Takagi H, Fujibe T, Aya K, Kojima M, Sakakibara H, Uemura A, Matsuoka M, Terauchi R (2012). *OsGA20ox1*, a candidate gene for a major QTL controlling seedling vigor in rice. Theor Appl Genet.

[CR2] Alexander L (2002). Ethylene biosynthesis and action in tomato: a model for climacteric fruit ripening. J Exp Bot.

[CR3] Anandan A, Anumalla M, Pradhan SK, Ali J (2016) Population structure, diversity and trait association analysis in rice (*Oryza sativa* L.) germplasm for early seedling vigor (ESV) using trait linked SSR markers. PLoS One 11(3):e0152406. 10.1371/journal.pone.0152406 10.1371/journal.pone.0152406 PMC4816567 27031620

[CR4] Anandan A, Mahender A, Sah RP, Bose LK, Subudhi H, Meher J, Reddy JN, Ali J (2020). Non-destructive phenotyping for early seedling vigor in direct-seeded rice. Plant Methods.

[CR5] Anandan A, Panda S, Sabarinathan S, Travis AJ, Norton GJ, Price AH (2022) Superior haplotypes for early root vigor traits under dry direct seeded low nitrogen condition through Genome Wide Association Mapping. Frontiers in plant Science 13:911775. 10.3389/fpls.2022.911775 10.3389/fpls.2022.911775 PMC9305665 35874029

[CR6] Arshad MS, Farooq M, Asch F, Krishna JSV, Prasad PVV, Siddique KHM (2017). Thermal stress impacts reproductive development and grain yield in rice. Plant Physiol Biochem.

[CR7] Asano K, Takashi T, Miura K (2007). Genetic and molecular analysis of utility of *sd1* alleles in rice breeding. Breed Sci.

[CR8] Ayano M, Kani T, Kojima M, Sakakibara H, Kitaoka T, Kuroha T, Angeles-Shim RB, Kitano H, Nagai K, Ashikari M (2014). Gibberellin biosynthesis and signal transduction is essential for internode elongation in deepwater rice. Plant Cell Environ.

[CR9] Bhullar MS, Kumar S, Kaur S, Kaur T, Singh J, Yadav R, Gill G (2016). Management of complex weed flora in dry-seeded rice. Crop Prot.

[CR10] Bian HW, Xie YK, Guo F, Han N, Ma SY, Zeng ZH, Wang JH, Yang YN, Zhu MY (2012). Distinctive expression patterns and roles of the *miRNA393/TIR1* homolog module in regulating flag leaf inclination and primary and crown root growth in rice (*Ory za sativa*). New Phytol.

[CR11] Biswas PS, Khatun H, Das N, Sarker MM, Anisuzzaman M (2017) Mapping and validation of QTLs for cold tolerance at seedling stage in rice from an indica cultivar Habiganj Boro VI (Hbj.BVI). 3 Biotech 7(6):359. 10.1007/s13205-017-0993-1 10.1007/s13205-017-0993-1 PMC5626667 28979832

[CR12] Catolos M, Sandhu N, Dixit S, Shamsudin NAA, Naredo MEB, McNally KL, Henry A, Diaz MG, Kumar A (2017). Genetic loci governing grain yield and root development under variable rice cultivation conditions. Front Plant Sci.

[CR13] Chakraborty D, Ladha JK, Rana DS, Jat ML, Gathala MK, Yadav S, Raman A (2017). A global analysis of alternative tillage and crop establishment practices for economically and environmentally efficient rice production. Sci Rep.

[CR14] Chauhan BS (2013). Strategies to manage weedy rice in Asia. Crop Prot.

[CR15] Chauhan BS, Abugho SB (2013). Effects of water regime, nitrogen fertilization, and rice plant density on growth and reproduction of lowland weed Echinochloa crus-galli. Crop Protoc.

[CR16] Chauhan BS, Awan TH, Abugho SB, Evengelista G (2015). Effect of crop establishment methods and weed control treatments on weed management, and rice yield. Field Crops Res.

[CR17] Chen K, Zhang Q, Wang CC, Liu ZX, Jiang YJ, Zhai LY, Zheng TQ, Xu JL, Li ZK (2019) Genetic dissection of seedling vigour in a diverse panel from the 3,000 Rice (*Oryza sativa* L.) Genome Project. Sci Rep (1):4804. 10.1038/s41598-019-41217-x 10.1038/s41598-019-41217-x PMC6423299 30886215

[CR18] Choi D, Lee Y, Cho HT, Kende H (2003). Regulation of expansin gene expression affects growth and development in transgenic rice plants. Plant Cell.

[CR19] Courtois B, Ahmadi N, Khowaja F, Price AH, Rami JF, Frouin J, Hamelin C, Ruiz M (2009). Rice root genetic architecture: meta-analysis from a drought QTL database. Rice.

[CR20] Crepin N, Rolland F (2019). *SnRK1* activation, signaling, and networking for energy homeostasis. Curr Opin Plant Biol.

[CR21] Dang X, Thi TG, Dong G, Wang H, Edzesi WM, Hong D (2014) Genetic diversity and association mapping of seed vigor in rice (*Oryza sativa,*L.). Planta 239(6):1309–1319. 10.1007/s00425-014-2060-z 10.1007/s00425-014-2060-z 24668487

[CR22] Dilday RH, Mgonja MA, Amonsilpa SA, Collins FC, Wells BR (1990) Plant height vs. mesoeotyl and elongation in rice: Linkage or pleioutropism? Crop Science 30(4):815–818. 10.2135/cropsci1990.0011183X003000040010x

[CR23] Egli DB, Rucker M (2012). Seed vigor and the uniformity of emergence of corn seedlings. Crop Sci.

[CR24] Farooq M, Basra SMA, Wahid A (2006). Priming of field-sown rice seed enhances germination, seedling establishment, allometry and yield. Plant Growth Regul.

[CR25] Farooq M, Siddique KHM, Rehman H, Aziz T, Lee DJ (2011). Rice direct seeding: Experiences, challenges and opportunities. Soil and Tillage Res.

[CR26] Finch-Savage WE, Bassel GW (2016) Seed vigour and crop establishment: extending performance beyond adaptation. J Exp Bot 67(3):567–591. 10.1093/jxb/erv490 10.1093/jxb/erv490 26585226

[CR27] Finch-Savage WE, Clay HA, Lynn JR, Morris K (2010). Towards a genetic understanding of seed vigour in small-seeded crops using natural variation in Brassica oleracea. Plant Sci.

[CR28] Foolad MR, Subbiah P, Zhang L (2007). Common QTL affect the rate of tomato seed germination under different stress and non stress conditions. Int J Plant Genomics.

[CR29] Fujino K, Sekiguchi H (2011). Origins of functional nucleotide polymorphisms in a major quantitative trait locus, *qLTG3-1*, controlling low-temperature germinability in rice. Plant Mol Biol.

[CR30] Fujino K, Sekiguchi H, Matsuda Y, Sugimoto K, Ono K, Yano M (2008). Molecular identification of a major quantitative trait locus, *qLTG3–1*, controlling low-temperature germinability in rice. Proc Natl Acad Sci.

[CR31] Fujino K, Sekiguchi H, Sato T, Kiuchi H, Nonoue Y, Takeuchi Y, Ando T, Lin SY, Yano M (2004) Mapping of quantitative trait loci controlling low-temperature germinability in rice (*Oryza sativa* L.) Theor Appl Genet 108(5):794–799. 10.1007/s00122-003-1509-4 10.1007/s00122-003-1509-4 14624339

[CR32] Gao SP, Fang J, Xu F, Wang W, Sun XH, Chu JF, Cai BD, Feng YQ, Chu CC (2014). *CYTOKININ OXIDASE/DEHYDROGENASE4* Integrates Cytokinin and Auxin Signaling to Control Rice Crown Root Formation. Plant Physiol.

[CR33] Gommers CMM, Monte E (2018). Seedling establishment: a dimmer switch-regulated process between dark and light signaling. Plant Physiol.

[CR34] Guglielminetti L, Perata P, Alpi A (1995). Effect of anoxia on carbohydrate metabolism in rice seedlings. Plant Physiol.

[CR35] Guo F, Han N, Xie YK, Fang K, Yang YN, Zhu MY, Wang JH, Bian HW (2016) The *miR393a*/target module regulates seed germination and seedling establishment under submergence in rice (*Oryza sativa* L.). Plant Cell Environ 39(10):2288–2302. 10.1111/pce.12781 10.1111/pce.12781 27342100

[CR36] Gupta K, Kumar R, Baruah KK, Hazarika S, Karmakar S, Bordoloi N (2021). Greenhouse gas emission from rice fields: a review from Indian context. Environ Sci Pollut Res Int.

[CR37] Hanzawa E, Sasaki K, Nagai S, Obara M, Fukuta Y, Uga Y, Miyao A, Hirochika H, Higashitani A, Maekawa M, Sato T (2013) Isolation of a novel mutant gene for soil-surface rooting in rice (Oryza sativa L.). Rice 6(1):30. 10.1186/1939-8433-6-30 10.1186/1939-8433-6-30 PMC3874653 24280269

[CR38] Hattori Y, Nagai K, Furukawa S, Song X, Kawano R, Sakakibara H, Wu J, Matsumoto T, Yoshimura A, Kitano H, Matsuoka M, Mori H, Ashikari M (2009). The ethylene response factors *SNORKEL1* and *SNORKEL2* allow rice to adapt to deep water. Nature.

[CR39] He Y, Chen SS, Liu KX, Chen YJ, Cheng YH, Zeng P, Zhu PW, Xie T, Chen SL, Zhang HS, Cheng JP (2022). OsHIPL 1, a hedgehog-interacting protein-like 1 protien, increases seed vigor in rice. Plant Biotechnol J.

[CR40] He Y, Yang B, He Y, Zhan C, Cheng Y, Zhang J, Zhang H, Cheng J, Wang Z (2019). A quantitative trait locus *qSE3* promotes seed germination and seedling establishment under salinity stress in rice. Plant J.

[CR41] He YQ, Cheng JP, He Y, Yang B, Cheng YH, Yang C, Zhang HS, Wang ZF (2019). Influence of isopropylmalate synthase *OsIPMS1* on seed vigour associated with amino acid and energy metabolism in rice. Plant Biotechnol J.

[CR42] Heredia MC, Kant J, Prodhan MA, Dixit S, Wissuwa M (2021). Breeding rice for a changing climate by improving adaptations to water saving technologies. Theor Appl Genet.

[CR43] Hickey LT, Hafeez AN, Robinson H, Jackson SA, Leal-bertioli SCM, Tester M, Gao CX, Godwin ID, Hayes BJ, Wulff BBH (2019). Breeding crops to feed 10 billion. Nat Biotechnol.

[CR44] Huang ZB, Ying JF, Peng LL, Sun S, Huang CW, Li C, Wang ZF, He YQ (2021). A genome-wide association study reveals that the cytochrome b5 involved in seed reserve mobilization during seed germination in rice. Theor Appl Genet.

[CR45] Hussain S, Huang J, Huang J, Ahmad S, Nanda S, Anwar S, Shakoor S, Zhu CQ, Zhu LF, Cao XC, Jin QY, Zhang JH (2020) Rice production under climate change: Adaptations and mitigating strategies. In: Environment, climate, plant and vegetation growth. Springer, Cham, pp 659–686. 10.1007/978-3-030-49732-3_26

[CR46] Hwang YS, Thomas BR, Rodriguez RL (1999). Differential expression of rice α-amylase genes during seedling development under anoxia. Plant Mol Biol.

[CR47] Ismail AM, Ella ES, Vergara GV, Mackill DJ (2009). Mechanisms associated with tolerance to flooding during germination and early seedling growth in rice (*Oryza sativa*). Ann Bot.

[CR48] Kaur K, Kaur P, Kaur T (2017). Problems faced by farmers in cultivation of direct seeded rice in Indian Punjab. Agric Res J.

[CR49] Kazan K (2015). Diverse roles of jasmonates and ethylene in abiotic stress tolerance. Trends Plant Sci.

[CR50] Keller M, Karutz Ch, Schmid JE, Stamp P, Winzeler M, Keller B, Messmer MM (1999). Quantitative trait loci for lodging resistance in a segregating wheat × spelt population. Theor Appl Genet.

[CR51] Kitomi Y, Kanno N, Kawai S, Mizubayashi T, Fukuoka S, Uga Y (2015). QTLs underlying natural variation of root growth angle among rice cultivars with the same functional allele of *DEEPER ROOTING 1*. Rice.

[CR52] Kordan HA (1974). Patterns of shoot and root growth in rice seedlings germinating under water. J Appl Ecol.

[CR53] Kretzschmar T, Pelayo MA, Trijatmiko KR, Gabunada LF, Alam R, Jimenez R, Mendioro MS, Slamet-Loedin IH, Sreenivasulu N, Bailey-Serres J, Ismail AM, Mackill DJ, Septiningsih EM (2015). A trehalose-6-phosphate phosphatase enhances anaerobic germination tolerance in rice. Nat Plants.

[CR54] Kuai J, Yang Y, Sun YY, Zhou GS, Zuo QS, Wu JS, Ling XX (2015). Paclobutrazol increases canola seed yield by enhancing lodging and pod shatter resistance in Brassica napus L. Field Crop Res.

[CR55] Kumar V, Ladha JK (2011). Direct seeding of rice: recent developments and future research needs. Adv Agron.

[CR56] Kuroha T, Nagai K, Gamuyao R, Wang DR, Furuta T, Nakamori M, Kitaoka T, Adachi K, Minami A, Mori Y (2018). Ethylene-gibberellin signaling underlies adaptation of rice to periodic flooding. Science.

[CR57] Lasanthi-Kudahettige R, Magneschi L, Loreti E, Gonzali S, Licausi F, Novi G, Beretta O, Vitulli F, Alpi A, Perata P (2007). Transcript profiling of the anoxic rice coleoptile. Plant Physiol.

[CR58] Lee HS, Sasaki K, Kang JW, Sato T, Song WY, Ahn SN (2017). Mesocotyl elongation is essential for seedling emergence under deep-seeding condition in rice. Rice.

[CR59] Lee KW, Chen PW, Lu CA, Chen S, Ho TH, Yu SM (2009) Coordinated responses to oxygen and sugar deficiency allow rice seedlings to tolerate flooding. Sci Signal 2(91):ra61. 10.1126/scisignal.2000333 10.1126/scisignal.2000333 19809091

[CR60] Li J, Han Y, Liu L, Chen Y, Du Y, Zhang J, Sun H, Zhao Q (2015). *qRT9*, a quantitative trait locus controlling root thickness and root length in upland rice. J Exp Bot.

[CR61] Li WJ, Yang B, Xu JY, Peng LL, Sun S, Huang ZB, Jiang XH, He YQ, Wang ZF (2021). A genome-wide association study reveals that the 2-oxoglutarate/malate translocator mediates seed vigor in rice. Plant J.

[CR62] Li XY, Qin FH, Chi ZZ, Jiang XL, Guo X, Zheng JG, Liu SR (2017). Study on Water Managemnent for Mechanical Precise Hill-drop Drilling Rice during Emergence Satge. Southwest China J Agric Sci.

[CR63] Liang Q, Wang C, Dr Ma, Li L, Cui ZB, Wang XX, Qian Q, Cai BD, Feng YQ, Chen WF (2016). Cortical microtubule disorganized related to an endogenous gibberellin increase plays an important role in rice mesocotyl elongation. Plant Biotechnol.

[CR64] Liu C, Zheng S, Gui JS, Fu CJ, Yu HS, Song DL, Shen JH, Qin P, Liu XM, Han B, Yang YZ, Li LG (2018). Shortened basal internodes encodes a gibberellin 2-oxidase and contributes to lodging resistance in rice. Mol Plant.

[CR65] Liu S, Huang Y, Xu H, Zhao M, Xu Q, Li F (2018). Genetic enhancement of lodging resistance in rice due to the key cell wall polymer lignin, which affects stem characteristics. Breed Sci.

[CR66] Loreti E, Alpi A, Perata P (2003). α-amylase expression under anoxia in rice seedlings: An update. Russ J Plant Physiol.

[CR67] Luo QY (2011). Temperature thresholds and crop production: a review. Clim Change.

[CR68] Lv YS, Shao GN, Jiao GA, Sheng ZH, Xie LH, Hu SK, Tang SQ, Wei XG, Hu PS (2021). Targeted mutagenesis of *POLYAMINE OXIDASE 5* that negatively regulates mesocotyl elongation enables the generation of direct-seeding rice with improved grain yield. Mol Plant.

[CR69] Ma YM, Wang J, Yang TF, Dong JF, Yang W, Chen L, Zhou L, Chen JS, Liu B, Zhang SH, Edwards D, Zhao JL (2022) Genome-wide association mapping and gene expression analysis identify *OsCPS1* as a new candidate gene controlling early seedling length in rice. Front Plant Sci 13:976669. 10.3389/fpls.2022.976669 10.3389/fpls.2022.976669 PMC9478204 36119573

[CR70] Ma YM, Zhao JL, Fu H, Yang TF, Dong JF, Yang W, Chen L, Zhou L, Wang J, Liu B, Zhang SH, Edwards D (2021). Genome-wide identification, expression and functional analysis reveal the involvement of FCS-like zinc finger gene family in submergence response in rice. Rice.

[CR71] Mahender A, Anandan A, Pradhan SK (2015). Early seedling vigour, an imperative trait for direct-seeded rice: an overview on physio-morphological parameters and molecular markers. Planta.

[CR72] Mao CJ, He JM, Liu L, Deng QM, Yao XF, Liu CM, Qiao YL, Li P, Ming F (2020). *OsNAC2* integrates auxin and cytokinin pathways to modulate rice root development. Plant Biotechnol J.

[CR73] Meng F, Xiang D, Zhu J, Li Y, Mao C (2019). Molecular mechanisms of root development in rice. Rice.

[CR74] Minic Z (2008). Physiological roles of plant glycoside hydrolases. Planta.

[CR75] Minic Z, Jouanin L (2006). Plant glycoside hydrolases involved in cell wall polysaccharide degradation. Plant Physiol Biochem.

[CR76] Mulsanti IW, Yamamoto T, Ueda T, Samadi AF, Kamahora E, Rumanti IA, Thanh VC, Adachi S, Suzuki S, Kanekatsu M, Hirasawa T, Ookawa T (2018). Finding the superior allele of japonica-type for increasing stem lodging resistance in indica rice varieties using chromosome segment substitution lines. Rice.

[CR77] Najeeb S, Mahender A, Anandan A, Hussain W, Li ZK, Ali J (2021) Genetics and breeding of low-temperature stress tolerance in rice. Rice Improv 221–280. 10.1007/978-3-030-66530-2_8

[CR78] Narsai R, Edwards JM, Roberts TH, Whelan J, Joss GH, Atwell BJ (2015). Mechanisms of growth and patterns of gene expression in oxygen-deprived rice coleoptiles. Plant J.

[CR79] Nguyen NV, Ferrero A (2006). Meeting the challenges of global rice production. Paddy Water Environ.

[CR80] Niroula RK, Pucciariello C, Ho VT, Novi G, Fukao T, Perata P (2012). *SUB1A*-dependent and-independent mechanisms are involved in the flooding tolerance of wild rice species. Plant J.

[CR81] Nomura T, Arakawa N, Yamamoto T, Ueda T, Adachi S, Yonemaru JI, Abe A, Takagi H, Yokoyama T, Ookawa T (2019) Next generation long-culm rice with superior lodging resistance and high grain yield, monster rice 1. PLoS One 14(8):e0221424. 10.1371/journal.pone.0221424 10.1371/journal.pone.0221424 PMC6705783 31437205

[CR82] Ookawa T, Hobo T, Yano M, Murata K, Ando T, Miura H, Asano K, Ochiai Y, Ikeda M, Nishitani R, Ebitani T, Ozaki H, Angeles ER, Hirasawa T, Matsuoka M (2010). New approach for rice improvement using a pleiotropic QTL gene for lodging resistance and yield. Nat Commun.

[CR83] Ookawa T, Ishihara K (1992). Varietal difference of physical characteristics of the culm related to lodging resistance in paddy rice. Jpn J Crop Sci.

[CR84] Otomo K, Kenmoku H, Oikawa H, König WA, Toshima H, Mitsuhashi W, Yamane H, Sassa T, Toyomasu T (2004). Biological functions of ent- and syn-copalyl diphosphate synthases in rice: key enzymes for the branch point of gibberellin and phytoalexin biosynthesis. Plant J.

[CR85] Pan JF, Zhao JL, Liu YZ, Huang NR, Tian K, Shah F, Liang KM, Zhong XH, Liu B (2019). Optimized nitrogen management enhances lodging resistance of rice and its morpho-anatomical, mechanical, and molecular mechanisms. Sci Rep.

[CR86] Peng L, Sun S, Yang B, Zhao J, Li W, Huang Z, Li Z, He Y, Wang Z (2021a) Genome-wide association study reveals that the cupin domain protein *OsCDP3.10* regulates seed vigour in rice. Plant Biotechnol J 20(3):485–498. 10.1111/pbi.13731 10.1111/pbi.13731 PMC8882794 34665915

[CR87] Peng S, Khush GS (2003). Four decades of breeding for varietal improvement of irrigated lowland rice in the International Rice Research Institute. Plant Prod Sci.

[CR88] Peng SB, Tang QY, Zou YB (2009). Current status and challenges of rice production in China. Plant Prod Sci.

[CR89] Peng Y, Hu YG, Qian Q, Ren DY (2021). Progress and Prospect of Breeding Utilization of Green Revolution Gene *SD 1* in Rice. Agriculture.

[CR90] Perata P, Pozueta-Romero J, Akazawa T, Yamaguchi J (1992). Effect of anoxia on starch breakdown in rice and wheat seeds. Planta.

[CR91] Prisic S, Xu M, Wilderman PR, Peters RJ (2004). Rice contains two disparate ent-copalyl diphosphate synthases with distinct metabolic functions. Plant Physiol.

[CR92] Pucciariello C (2020). Molecular mechanisms supporting rice germination and coleoptile elongation under low oxygen. Plants.

[CR93] Rajjou L, Duval M, Gallardo K, Catusse J, Bally J, Job C, Job D (2012). Seed germination and vigor. Annu Rev Plant Biol.

[CR94] Rao AN, Johnson DE, Sivaprasad B, Ladha JK, Mortimer AM (2007). Weed management in direct-seeded rice. Adv Agron.

[CR95] Redoña ED, Mackill DJ (1996). Mapping quantitative trait loci for seeding vigor in rice using RFLPs. Theor Appl Genet.

[CR96] Sakamoto T, Miura K, Itoh H, Tatsumi T, Ueguchi-Tanaka M, Ishiyama K, Kobayashi M, Agrawal GK, Takeda S, Abe K, Miyao A, Hirochika H, Kitano H, Ashikari M, Matsuoka M (2004). An overview of gibberellin metabolism enzyme genes and their related mutants in rice. Plant Physiol.

[CR97] Savary S, Castilla NP, Elazegui FA, Teng PS (2005). Multiple effects of two drivers of agricultural change, labour shortage and water scarcity, on rice pest profiles in tropical Asia. Field Crops Res.

[CR98] Setter TL, Laureles EV, Mazaredo AM (1997). Lodging reduces yield of rice by self shading and reduction of photosynthesis. Field Crops Res.

[CR99] Shah L, Yahya M, Shah SMA, Nadeem M, Ali A, Ali A, Wang J, Riaz MW, Rehman S, Wu W, Khan RM, Abbas A, Riaz A, Anis GB, Si H, Jiang H, Ma C (2019). Improving lodging resistance: using wheat and rice as classical examples. Int J Mol Sci.

[CR100] Shim KC, Kim SH, Lee HS, Adeva C, Jeon YA, Luong NH, Kim WJ, Akhtamov M, Park YJ, Ahn SN (2020). Characterization of a new *qLTG3–1* allele for low-temperature germinability in rice from the wild species Oryza rufipogon. Rice.

[CR101] Shirasawa S, Endo T, Nakagomi K, Yamaguchi M, Nishio T (2012). Delimitation of a QTL region controlling cold tolerance at booting stage of a cultivar, 'Lijiangxintuanheigu', in rice, *Oryza sativa* L. Theor Appl Genet.

[CR102] Shrestha S, Laza MRC, Mendez KV, Bhosale S, Dingkuhn M (2020) The blaster: a methodology to induce rice lodging at plot scale to study lodging resistance. Field Crop Res 245:107663. 10.1016/j.fcr.2019.107663

[CR103] Si-Ammour A, Windels D, Arn-Bouldoires E, Kutter C, Ailhas J, Meins F, Vazquez F (2011). *miR393* and secondary siRNAs regulate expression of the *TIR1/AFB2* auxin receptor clade and auxin-related development of Arabidopsis leaves. Plant Physiol.

[CR104] Singh S, Sharma SN, Prasad R (2001). The effect of seeding and tillage methods on productivity of rice–wheat cropping system. Soil Tillage Res.

[CR105] Singh UM, Yadav S, Dixit S, Ramayya PJ, Devi MN, Raman KA, Kumar A (2017) QTL Hotspots for Early Vigor and Related Traits under Dry Direct-Seeded System in Rice (*Oryza sativa* L.). Front Plant Sci 8:286. 10.3389/fpls.2017.00286 10.3389/fpls.2017.00286 PMC5332406 28303149

[CR106] Sun J, Zhang GC, Cui ZB, Kong XM, Yu XY, Gui R, Han YQ, Li Z, Lang H, Hua YC, Zhang XM, Xu Q, Tang L, Xu ZJ, Ma DR, Chen WF (2022). Regain flood adaptation in rice through a 14–3-3 protein *OsGF14h*. Nat Commun.

[CR107] Sun SY, Wang T, Wang LL, Li XM, Jia YC, Liu C, Huang XH, Xie WB, Wang XL (2018). Natural selection of a *GSK3* determines rice mesocotyl domestication by coordinating strigolactone and brassinosteroid signaling. Nat Commun.

[CR108] Tao Y, Chen Q, Peng S, Wang W, Nie L (2016). Lower global warming potential and higher yield of wet direct-seeded rice in Central China. Agron Sustain Dev.

[CR109] Tirol-Padre A, Rai M, Kumar V, Gathala M, Sharma PC, Sharma S, Nagar KR Deshwal S, Singh LK, Jat HS, Sharma DK, Wassmann R, Ladha J (2016) Quantifying changes to the global warming potential of rice-wheat systems with the adoption of conservation agriculture in northwestern India. Agric Ecosyst Environ 219:125–13710.1016/j.agee.2015.12.020

[CR110] Tu B, Tao Z, Wang S, Zhou L, Zheng L, Zhang C, Li X, Zhang X, Yin J, Zhu X, Yuan H, Li T, Chen W, Qin P, Ma B, Wang Y, Li S (2022). Loss of *Gn1a/OsCKX2* confers heavy-panicle rice with excellent lodging resistance. J Integr Plant Biol.

[CR111] Uga Y, Hanzawa E, Nagai S, Sasaki K, Yano M, Sato T (2012). Identification of *qSOR1*, a major rice QTL involved in soil-surface rooting in paddy fields. Theor Appl Genet.

[CR112] Wang FM, Longkumer T, Catausan SC, Calumpang CLF, Tarun JA, Cattin-Ortola J, Ishizaki T, Pariasca Tanaka J, Rose T, Wissuwa M, Kretzschmar T (2018). Genome-wide association and gene validation studies for early root vigour to improve direct seeding of rice. Plant Cell Environ.

[CR113] Wang WX, Du J, Zhou YZ, Zeng YJ, Tan XM, Pan XH, Shi QH, Wu ZM, Zeng YH (2021). Effects of different mechanical direct seeding methods on grain yield and lodging resistance of early indica rice in South China. J Integr Agric.

[CR114] Wang X, Zou BH, Shao QL, Cui YM, Lu S, Zhang Y, Huang QS, Huang J, Hua J (2018). Natural variation reveals that *OsSAP16* controls low-temperature germination in rice. J Exp Bot.

[CR115] Wang Y, Wang YT, Yang RF, Wang FH, Fu J, Yang WB, Bai T, Wang SX, Yin HQ (2021). Effects of gibberellin priming on seedling emergence and trans involved in mesocotyl elongation in rice under deep direct-seeding conditions. J Zhejiang Univ Sci B.

[CR116] Wang ZF, Wang JF, Bao YM, Wang FH, Zhang HS (2010). Quantitative trait loci analysis for rice seed vigor during the germination stage. J Zhejiang Univ Sci B.

[CR117] Woo YM, Park HJ, Su'udi M, Yang JI, Park JJ, Back K, Park YM, An G (2007). Constitutively wilted 1, a member of the rice YUCCA gene family, is required for maintaining water homeostasis and an appropriate root to shoot ratio. Plant Mol Biol.

[CR118] Wu YX, Guo CC, Sun YJ, Liu FY, Zhang Q, Xiang KH, Sun YY, Ma J (2020) Relationship of population quality and nitrogen fertilizer utilization characteristics of direct seeding rice under water-nitrogen interaction. Ying Yong Sheng Tai Xue Bao 31(3):899–908. 10.13287/j.1001-9332.202003.022 10.13287/j.1001-9332.202003.022 32537986

[CR119] Xia K, Wang R, Qu X, Fang Z, Tian C, Duan J, Wang Y, Zhang M (2012) *OsTIR1* and *OsAFB2* downregulation via *OsmiR393* overexpression leads to more tillers, early flowering and less tolerance to salt and drought in rice. PLoS One 7:e30039. 10.1371/journal.pone.0030039 10.1371/journal.pone.0030039 PMC3254625 22253868

[CR120] Xie LX, Tan ZW, Zhou Y, Xu RB, Feng LB, Xing YZ, Qi XQ (2014). Identification and fine mapping of quantitative trait loci for seed vigor in germination and seedling establishment in rice. J Integr Plant Biol.

[CR121] Xiong Q, Ma B, Lu X, Huang YH, He SJ, Yang C, Yin CC, Zhao H, Zhou Y, Zhang WK, Wang WS, Li ZK, Chen SY, Zhang JS (2017). Ethylene-inhibited jasmonic acid biosynthesis promotes mesocotyl/coleoptile elongation of etiolated rice seedlings. Plant Cell.

[CR122] Xu L, LI XX, Wang XY, Xiong DL, Wang F (2019) Comparing the grain yields of direct-seeded and transplanted rice: a meta-analysis. Agronomy 9(11):76710.3390/agronomy9110767

[CR123] Yadav S, Gill G, Humphreys E, Kukal SS, Walia US (2011). Effect of water management on dry seeded and puddled transplanted rice: Part 2: water balance and water productivity. Field Crop Res.

[CR124] Yadav S, Singh UM, Naik SM, Venkateshwarlu C, Ramayya PJ, Raman KA, Sandhu N, Kumar A (2017). Molecular mapping of QTLs associated with lodging resistance in dry direct-seeded rice (*Oryza sativa* L.). Front Plant Sci.

[CR125] Yang J, Guo ZH, Luo LX, Gao QL, Xiao WM, Wang JF, Wang H, Chen ZQ, Guo T (2021). Identification of QTL and candidate genes involved in early seedling growth in rice via high-density genetic mapping and RNA-seq. Crop J.

[CR126] Yang TF, Zhou L, Zhao JL, Dong JF, Liu Q, Fu H, Mao XX, Yang W, Ma YM, Chen L, Wang J, Bai S, Zhang SH, Liu B (2020) The Candidate Genes Underlying a Stably Expressed QTL for Low Temperature Germinability in Rice (*Oryza sativa* L.). Rice (N Y) 13(1):74. 10.1186/s12284-020-00434-z 10.1186/s12284-020-00434-z PMC7573065 33074410

[CR127] Yu SM, Lee HT, Lo SF, Ho TD (2021). How does rice cope with too little oxygen during its early life?. New Phytol.

[CR128] Zeng DL, Tian ZX, Rao YC, Dong GJ, Yang YL, Huang LC, Leng YJ, Xu J, Sun C, Zhang GH, Hu J, Zhu L, Gao ZY, Hu XM, Guo LB, Xiong GS, Wang YH, Li JY, Qian Q (2017). Rational design of high-yield and superior-quality rice. Nat Plants.

[CR129] Zhan JH, Lu X, Liu HY, Zhao QZ, Ye GY (2020) Mesocotyl elongation, an essential trait for dry-seeded rice (*Oryza sativa* L.): a review of physiological and genetic basis. Planta 251(1):27. 10.1007/s00425-019-03322-z 10.1007/s00425-019-03322-z 31802259

[CR130] Zhang A, Liu C, Chen G, Hong K, Gao Y, Tian P, Peng Y, Zhang B, Ruan B, Jiang H, Guo L, Qian Q, Gao Z (2017). Genetic analysis for rice seedling vigor and fine mapping of a major QTL *qSSL1b* for seedling shoot length. Breed Sci.

[CR131] Zhang Q, Chen QH, Wang SL, Hong Y, Wang Z (2014). Rice and cold stress: methods for its evaluation and summary of cold tolerance-related quantitative trait loci. Rice (N Y).

[CR132] Zhang WJ, Wu LM, Ding YF, Wen F, Wu XR, Li GH, Liu ZH, Tang S, Ding CQ, Wang SH (2016). Top-dressing nitrogen fertilizer rate contributes to decrease culm physical strength by reducing structural carbohydrate content in japonica rice. J Integr Agric.

[CR133] Zhao DL, Atlin GN, Bastiaans L, Spiertz JH (2006). Comparing rice germplasm groups for growth, grain yield and weed-suppressive ability under aerobic soil conditions. Weed Res.

[CR134] Zhao J, He YQ, Huang SL, Wang ZF (2021a) Advances in the Identification of Quantitative Trait Loci and Genes Involved in Seed Vigor in Rice. Frontiers in Plant Science 12:659307. 10.3389/fpls.2021.659307 10.3389/fpls.2021.659307 PMC8316977 34335643

[CR135] Zhao J, Li WJ, Sun S, Peng LL, Huang ZB, He YQ, Wang ZF (2021). Affiliations expand The rice Small Auxin-Up RNA Gene *OsSAUR33* regulates seed vigor via sugar pathway during early seed germination. Int J Mol Sci.

[CR136] Zhao J, Yang B, Li WJ, Sun S, Peng LL, Feng DF, Li L, Di H, He YQ, Wang ZF (2021). A genome-wide association study reveals that the glucosyltransferase *OsIAGLU* regulates root growth in rice. J Exp Bot.

[CR137] Zhao Y, Zhao WP, Jiang CH, Wang XN, Xiong HY, Todorovska EG, Yin ZG, Chen YF, Wang X, Xie JY, Pan YH, Rashid MAR, Zhang HL, Li JJ, Li ZC (2018). Genetic architecture and candidate genes for deep-sowing tolerance in rice revealed by non-syn GWAS. Front Plant Sci.

[CR138] Zhou W, Guo Z, Chen J, Jiang J, Hui DF, Wang X, Sheng J, Chen LG, Luo YQ, Zheng JC, Li SF, Zhang YF (2019) Direct seeding for rice production increased soil erosion and phosphorus runoff losses in subtropical China. Sci Total Environ 695:133845. 10.1016/j.scitotenv.2019.133845 10.1016/j.scitotenv.2019.133845 31421335

[CR139] Zhu LH, Zhong DB, Xu JL, Yu SB (2008) Differential expression of lodging resistance related QTL in rice (*Oryza sativa* L.). Plant Sci 175(6):898–905. 10.1016/j.plantsci.2008.09.001

[CR140] Zuber U, Winzeler H, Messmer MM, Keller M, Keller B, Schmid JE (1999) Morphological traits associated with lodging resistance of spring wheat *(Triticum aestivum* L.). J Agron Crop Sci 182(1):17–24. 10.1046/j.1439-037x.1999.00251.x

[CR141] Zuo QS, Kuai J, Zhao L, Hu Z, Wu JS, Zhou GS (2017). The effect of sowing depth and soil compaction on the growth and yield of rapeseed in rice straw returning field. Field Crop Res.

